# 2-Bromo-4-nitro­aniline

**DOI:** 10.1107/S1600536809004073

**Published:** 2009-02-06

**Authors:** Muhammad Nadeem Arshad, M. Nawaz Tahir, Islam Ullah Khan, Muhammad Shafiq

**Affiliations:** a Department of Chemistry, Government College University, Lahore, Pakistan; b Department of Physics, University of Sargodha, Sargodha, Pakistan

## Abstract

In the mol­ecule of the title compound, C_6_H_5_BrN_2_O_2_, the dihedral angle between the nitro group and the aromatic ring is 4.57 (4)°. An intra­molecular N—H⋯Br inter­action results in the formation of a planar five-membered ring, which is oriented with respect to the aromatic ring at a dihedral angle of 1.64 (6)°. In the crystal structure, inter­molecular N—H⋯N and N—H⋯O hydrogen bonds link the mol­ecules.

## Related literature

For related structures, see: Arshad *et al.* (2008 ▶, 2009 ▶); McPhail & Sim (1965 ▶); McWilliam *et al.* (2001 ▶); Krishna Mohan *et al.* (2004 ▶).
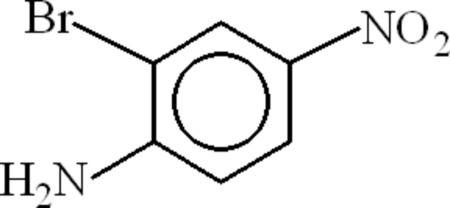


## Experimental

### 

#### Crystal data

C_6_H_5_BrN_2_O_2_
*M* *_r_* = 217.03
Orthorhombic, 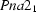

*a* = 11.098 (3) Å
*b* = 16.763 (4) Å
*c* = 3.9540 (9) Å
*V* = 735.6 (3) Å^3^
*Z* = 4
Mo *K*α radiation
μ = 5.53 mm^−1^
*T* = 296 (2) K
0.26 × 0.12 × 0.10 mm

#### Data collection

Bruker Kappa APEXII CCD diffractometer
Absorption correction: multi-scan (*SADABS*; Bruker, 2005 ▶) *T*_min_ = 0.450, *T*_max_ = 0.578
4932 measured reflections
1542 independent reflections
986 reflections with *I* > 2σ(*I*)
*R*_int_ = 0.058

#### Refinement

*R*[*F* ^2^ > 2σ(*F* ^2^)] = 0.039
*wR*(*F* ^2^) = 0.092
*S* = 1.00
1542 reflections
100 parameters
1 restraint
H-atom parameters constrained
Δρ_max_ = 0.50 e Å^−3^
Δρ_min_ = −0.62 e Å^−3^
Absolute structure: Flack (1983 ▶), 469 Friedel pairs
Flack parameter: 0.01 (2)

### 

Data collection: *APEX2* (Bruker, 2007 ▶); cell refinement: *SAINT* (Bruker, 2007 ▶); data reduction: *SAINT*; program(s) used to solve structure: *SHELXS97* (Sheldrick, 2008 ▶); program(s) used to refine structure: *SHELXL97* (Sheldrick, 2008 ▶); molecular graphics: *ORTEP-3 for Windows* (Farrugia, 1997 ▶) and *PLATON* (Spek, 2003 ▶); software used to prepare material for publication: *WinGX* (Farrugia, 1999 ▶) and *PLATON*.

## Supplementary Material

Crystal structure: contains datablocks global, I. DOI: 10.1107/S1600536809004073/hk2621sup1.cif

Structure factors: contains datablocks I. DOI: 10.1107/S1600536809004073/hk2621Isup2.hkl

Additional supplementary materials:  crystallographic information; 3D view; checkCIF report

## Figures and Tables

**Table 1:** Hydrogen-bond geometry (Å, °)

*D*—H⋯*A*	*D*—H	H⋯*A*	*D*⋯*A*	*D*—H⋯*A*
N1—H1*A*⋯N1^i^	0.86	2.32	3.158 (7)	167.00
N1—H1*B*⋯Br1	0.86	2.68	3.095 (5)	111.00
N1—H1*B*⋯O2^ii^	0.86	2.32	3.049 (7)	143.00

Symmetry codes: (i) 
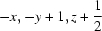
; (ii) 
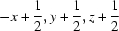
.

**Fig. 1.**
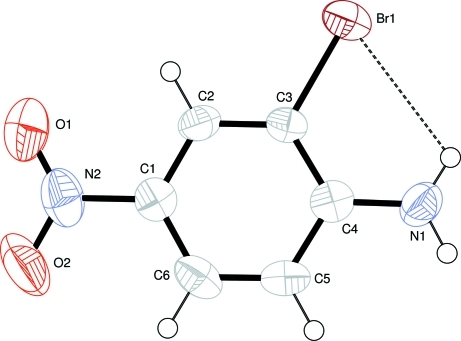
The molecular structure of the title molecule, with the atom-numbering scheme. Displacement ellipsoids are drawn at the 50% probability level. Hydrogen bond is shown as dashed line.

**Fig. 2.**
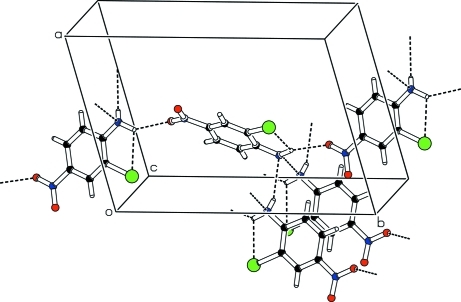
A partial packing diagram of the title compound. Hydrogen bonds are shown as dashed lines.

## supplementary crystallographic information

### Comment

The title compound, (I), has been prepared as an intermediate for the synthesis of sulfonamides (Arshad *et al.*, 2009) and benzothiazines (Arshad *et al.*, 2008).

The crystal structures of 2-iodo-4-nitroaniline, (II) (McWilliam *et al.*, 2001) and 2-chloro-4-nitroaniline, (III) (McPhail & Sim, 1965) have been reported. The title compound, (I), (Fig 1) is structural isomer of (III). It is essentially planar. The dihedral angle between the nitro group (O1/O2/N2) and the aromatic ring A (C1-C6) is 4.57 (4)°. The intramolecular N-H···Br interaction (Table 1) results in the formation of a planar five-membered ring (Br1/N1/C3/C4/H1B), which is oriented with respect to ring A at a dihedral angle of 1.64 (6)°. So, they are nearly coplanar.

In the crystal structure, intermolecular N-H···N and N-H···O hydrogen bonds (Table 1) link the molecules (Fig. 2), in which they may be effective in the stabilization of the structure.

### Experimental

The title compound was synthesized following the method available in literature (Krishna Mohan *et al.*, 2004). 4-Nitro aniline (6 g, 0.0435 mol) and ammonium bromide (4.5 g, 0.0479 mol) were charged to a flask (50 ml) containing acetic acid (30 ml). Hydrogen peroxide (1.629 g, 0.0479 mol, 35%) was added dropwise to the mixture, and stirred at room temperature for 3 h. Then, the obtained precipitate was filtered and washed with water and recrystallized in dichloromethane and methanol.

### Refinement

H-atoms were positioned geometrically, with N-H = 0.86 Å (for NH_2_) and C-H = 0.93 Å for aromatic H, and constrained to ride on their parent atoms, with U_iso_(H) = 1.2U_eq_(C, N).

### Crystal data

**Table:**

C_6_H_5_BrN_2_O_2_	*F*(000) = 424
*M**_r_* = 217.03	*D*_x_ = 1.960 Mg m^−^^3^
Orthorhombic, *P**n**a*2_1_	Mo *K*α radiation, λ = 0.71073 Å
Hall symbol: P 2c -2n	Cell parameters from 1542 reflections
*a* = 11.098 (3) Å	θ = 3.1–28.6°
*b* = 16.763 (4) Å	µ = 5.53 mm^−^^1^
*c* = 3.9540 (9) Å	*T* = 296 K
*V* = 735.6 (3) Å^3^	Needle, yellow
*Z* = 4	0.26 × 0.12 × 0.10 mm

### Data collection

**Table:**

Bruker Kappa APEXII CCD diffractometer	1542 independent reflections
Radiation source: fine-focus sealed tube	986 reflections with *I* > 2σ(*I*)
graphite	*R*_int_ = 0.058
Detector resolution: 7.40 pixels mm^-1^	θ_max_ = 28.6°, θ_min_ = 3.1°
ω scans	*h* = −13→14
Absorption correction: multi-scan (*SADABS*; Bruker, 2005)	*k* = −22→22
*T*_min_ = 0.450, *T*_max_ = 0.578	*l* = −3→5
4932 measured reflections	

### Refinement

**Table:**

Refinement on *F*^2^	Secondary atom site location: difference Fourier map
Least-squares matrix: full	Hydrogen site location: inferred from neighbouring sites
*R*[*F*^2^ > 2σ(*F*^2^)] = 0.039	H-atom parameters constrained
*wR*(*F*^2^) = 0.092	*w* = 1/[σ^2^(*F*_o_^2^) + (0.0302*P*)^2^] where *P* = (*F*_o_^2^ + 2*F*_c_^2^)/3
*S* = 1.00	(Δ/σ)_max_ = 0.001
1542 reflections	Δρ_max_ = 0.50 e Å^−^^3^
100 parameters	Δρ_min_ = −0.62 e Å^−^^3^
1 restraint	Absolute structure: Flack (1983), 469 Friedel pairs
Primary atom site location: structure-invariant direct methods	Flack parameter: 0.01 (2)

### Special details

**Table:**

Geometry. Bond distances, angles *etc*. have been calculated using the rounded fractional coordinates. All su's are estimated from the variances of the (full) variance-covariance matrix. The cell e.s.d.'s are taken into account in the estimation of distances, angles and torsion angles
Refinement. Refinement of *F*^2^ against ALL reflections. The weighted *R*-factor *wR* and goodness of fit *S* are based on *F*^2^, conventional *R*-factors *R* are based on *F*, with *F* set to zero for negative *F*^2^. The threshold expression of *F*^2^ > σ(*F*^2^) is used only for calculating *R*-factors(gt) *etc*. and is not relevant to the choice of reflections for refinement. *R*-factors based on *F*^2^ are statistically about twice as large as those based on *F*, and *R*- factors based on ALL data will be even larger.

### Fractional atomic coordinates and isotropic or equivalent isotropic displacement parameters (Å^2^)

**Table:**

	*x*	*y*	*z*	*U*_iso_*/*U*_eq_	
Br1	0.34261 (5)	0.56438 (3)	0.7237 (2)	0.0524 (2)	
O1	0.4834 (5)	0.2673 (3)	0.5214 (15)	0.088 (3)	
O2	0.3637 (4)	0.1882 (2)	0.763 (3)	0.101 (2)	
N1	0.1095 (4)	0.5118 (3)	1.0912 (13)	0.0510 (18)	
N2	0.3960 (5)	0.2544 (3)	0.689 (2)	0.064 (2)	
C1	0.3231 (4)	0.3212 (3)	0.8007 (18)	0.043 (3)	
C2	0.3615 (4)	0.3974 (3)	0.730 (3)	0.0420 (17)	
C3	0.2913 (5)	0.4600 (3)	0.8270 (13)	0.0347 (19)	
C4	0.1833 (5)	0.4491 (3)	0.9987 (15)	0.0377 (17)	
C5	0.1479 (5)	0.3705 (4)	1.0711 (16)	0.048 (2)	
C6	0.2169 (5)	0.3076 (3)	0.9745 (17)	0.051 (2)	
H1A	0.04332	0.50258	1.19738	0.0611*	
H1B	0.12976	0.55993	1.04260	0.0611*	
H2	0.43405	0.40604	0.61823	0.0502*	
H5	0.07627	0.36137	1.18676	0.0576*	
H6	0.19297	0.25579	1.02478	0.0605*	

### Atomic displacement parameters (Å^2^)

**Table:**

	*U*^11^	*U*^22^	*U*^33^	*U*^12^	*U*^13^	*U*^23^
Br1	0.0557 (3)	0.0436 (3)	0.0578 (4)	−0.0094 (3)	−0.0026 (7)	0.0100 (5)
O1	0.075 (4)	0.077 (4)	0.112 (5)	0.028 (3)	0.016 (4)	−0.009 (3)
O2	0.110 (4)	0.037 (2)	0.157 (6)	0.010 (2)	0.000 (5)	−0.006 (5)
N1	0.035 (2)	0.056 (3)	0.062 (4)	0.001 (2)	0.003 (2)	−0.008 (2)
N2	0.063 (3)	0.051 (3)	0.078 (5)	0.017 (3)	−0.018 (5)	−0.007 (5)
C1	0.041 (3)	0.046 (3)	0.041 (7)	0.001 (2)	−0.010 (3)	0.000 (3)
C2	0.034 (3)	0.049 (3)	0.043 (3)	−0.001 (2)	0.005 (5)	0.009 (6)
C3	0.034 (3)	0.037 (3)	0.033 (4)	−0.007 (2)	−0.007 (2)	0.002 (2)
C4	0.038 (3)	0.049 (3)	0.026 (3)	−0.002 (3)	−0.012 (3)	0.000 (3)
C5	0.043 (3)	0.053 (4)	0.047 (4)	−0.015 (3)	0.002 (3)	0.006 (3)
C6	0.056 (4)	0.037 (3)	0.059 (5)	−0.008 (3)	−0.018 (4)	0.005 (3)

### Geometric parameters (Å, °)

**Table:**

Br1—C3	1.885 (5)	C1—C2	1.375 (7)
O1—N2	1.195 (9)	C2—C3	1.362 (8)
O2—N2	1.202 (7)	C3—C4	1.390 (8)
N1—C4	1.382 (7)	C4—C5	1.404 (8)
N2—C1	1.450 (8)	C5—C6	1.358 (8)
N1—H1B	0.8600	C2—H2	0.9300
N1—H1A	0.8600	C5—H5	0.9300
C1—C6	1.383 (8)	C6—H6	0.9300
			
Br1···N1	3.095 (5)	C5···C1^ix^	3.576 (9)
Br1···H1B	2.6800	C5···C2^ix^	3.551 (11)
Br1···H2^i^	2.9700	C6···C1^ix^	3.480 (10)
O1···C6^ii^	3.391 (8)	C6···O1^x^	3.391 (8)
O2···N1^iii^	3.049 (7)	C4···H1A^v^	2.9000
O1···H2	2.4200	H1A···H5	2.4000
O1···H5^iv^	2.7300	H1A···N1^v^	2.9500
O2···H6	2.4400	H1A···N1^vi^	2.3200
O2···H1B^iii^	2.3200	H1A···C4^vi^	2.9000
N1···Br1	3.095 (5)	H1A···H1A^v^	2.2000
N1···N1^v^	3.158 (7)	H1A···H1A^vi^	2.2000
N1···N1^vi^	3.158 (7)	H1A···H1B^vi^	2.5800
N1···O2^vii^	3.049 (7)	H1B···Br1	2.6800
N1···H1A^v^	2.3200	H1B···H1A^v^	2.5800
N1···H1A^vi^	2.9500	H1B···O2^vii^	2.3200
C1···C5^viii^	3.576 (9)	H2···O1	2.4200
C1···C6^viii^	3.480 (10)	H2···Br1^xi^	2.9700
C2···C5^viii^	3.551 (11)	H5···H1A	2.4000
C3···C4^viii^	3.492 (8)	H5···O1^xii^	2.7300
C4···C3^ix^	3.492 (8)	H6···O2	2.4400
			
O1—N2—O2	123.0 (6)	C2—C3—C4	122.0 (5)
O1—N2—C1	118.8 (5)	N1—C4—C5	119.6 (5)
O2—N2—C1	118.2 (6)	N1—C4—C3	122.7 (5)
H1A—N1—H1B	120.00	C3—C4—C5	117.7 (5)
C4—N1—H1A	120.00	C4—C5—C6	120.9 (5)
C4—N1—H1B	120.00	C1—C6—C5	119.5 (5)
N2—C1—C6	120.0 (5)	C1—C2—H2	121.00
N2—C1—C2	118.8 (5)	C3—C2—H2	121.00
C2—C1—C6	121.2 (5)	C4—C5—H5	120.00
C1—C2—C3	118.8 (6)	C6—C5—H5	120.00
Br1—C3—C2	118.8 (4)	C1—C6—H6	120.00
Br1—C3—C4	119.2 (4)	C5—C6—H6	120.00
			
O1—N2—C1—C2	−4.5 (11)	C1—C2—C3—C4	0.9 (12)
O1—N2—C1—C6	175.4 (7)	Br1—C3—C4—N1	1.6 (8)
O2—N2—C1—C2	177.4 (9)	Br1—C3—C4—C5	179.9 (4)
O2—N2—C1—C6	−2.8 (11)	C2—C3—C4—N1	−178.3 (7)
N2—C1—C2—C3	178.2 (7)	C2—C3—C4—C5	0.1 (10)
C6—C1—C2—C3	−1.6 (13)	N1—C4—C5—C6	178.1 (6)
N2—C1—C6—C5	−178.5 (6)	C3—C4—C5—C6	−0.3 (9)
C2—C1—C6—C5	1.4 (11)	C4—C5—C6—C1	−0.4 (10)
C1—C2—C3—Br1	−179.0 (6)		

Symmetry codes: (i) −*x*+1, −*y*+1, *z*+1/2; (ii) *x*+1/2, −*y*+1/2, *z*; (iii) −*x*+1/2, *y*−1/2, *z*−1/2; (iv) *x*+1/2, −*y*+1/2, *z*−1; (v) −*x*, −*y*+1, *z*−1/2; (vi) −*x*, −*y*+1, *z*+1/2; (vii) −*x*+1/2, *y*+1/2, *z*+1/2; (viii) *x*, *y*, *z*−1; (ix) *x*, *y*, *z*+1; (x) *x*−1/2, −*y*+1/2, *z*; (xi) −*x*+1, −*y*+1, *z*−1/2; (xii) *x*−1/2, −*y*+1/2, *z*+1.

### Hydrogen-bond geometry (Å, °)

**Table:**

*D*—H···*A*	*D*—H	H···*A*	*D*···*A*	*D*—H···*A*
N1—H1A···N1^vi^	0.86	2.32	3.158 (7)	167.00
N1—H1B···Br1	0.86	2.68	3.095 (5)	111.00
N1—H1B···O2^vii^	0.86	2.32	3.049 (7)	143.00

Symmetry codes: (vi) −*x*, −*y*+1, *z*+1/2; (vii) −*x*+1/2, *y*+1/2, *z*+1/2.
